# Discoid lateral meniscus in children and adolescents: a histological study

**DOI:** 10.1186/s40634-018-0153-5

**Published:** 2018-09-24

**Authors:** Salvatore Bisicchia, Flavia Botti, Cosimo Tudisco

**Affiliations:** Dipartimento di Scienze Cliniche e, Medicina Traslazionale, Universita degli Studi di Roma Tor Vergata, Via Montpellier 1, 00133 Rome, Italy

**Keywords:** Discoid lateral meniscus, Histology, Children, Knee, Development

## Abstract

**Background:**

Discoid lateral meniscus is the most frequent variant of the meniscus. Although the histology of normal menisci in children and in adults has been well described, few studies have focused on the histology of discoid menisci. Furthermore, most of the patients in those studies were adults. The aim of the present study was to report the histological findings of discoid lateral meniscus in a group of children and adolescents, aged between 9 and 18, after arthroscopic partial resection, focusing on cellularity, arrangement of collagen fibers, and vascularity of the excised fragments. Furthermore, to report on MRI findings compared to the histological findings in the same region.

**Methods:**

Six patients (one female and five males) aged 9, 10, 13, 15, 17, and 18, were arthroscopically operated on partial meniscectomy (saucerization) of a discoid lateral meniscus, and the specimens were histologically examined.

**Results:**

The extracellular matrix showed a different distribution and characteristics depending on the different side of the meniscus. Irregularly oriented collagen fibers in discoid lateral meniscus were found. There were no blood vessels in the inner part of discoid lateral meniscus in all patients but the 18-year old (in which we observed also endothelials cells, edematous tissue and leaking of erythrocytes in the extracellular matrix). In the discoid lateral menisci analyzed, irregularly oriented collagen fibers with blood vessels were found only in the presence of degenerating tissue.

**Conclusions:**

Discoid lateral meniscus is different from a normal meniscus in terms of vascularity and disorganization of collagen fibers.

## Background

Discoid lateral meniscus is the most frequent variant of the meniscus and it is bilateral in up to 20% of cases (Yaniv & Blumberg, 2007). Although the histology of normal menisci in children and in adults has been well described (Clark & Ogden, 1983), few studies have focused on the histology of discoid menisci. Furthermore, most of the patients in those studies were adults (Atay et al., 2007; Papadopoulos et al., 2009; Cui & Min, 2007; Choi et al., 2017; Inoue et al., 2016). For these reasons, a description of the histology of discoid lateral meniscus in children and adolescents is warrant. As shown in a previous study, symptomatic discoid lateral meniscus has tear or degeneration, though no visible tear is observable at arthroscopy. Intra-meniscal degeneration can be detected at MRI examination (Hamada, 1994).

The aim of the present study was to report the histological findings of discoid lateral meniscus in a group of children and adolescents, aged between 9 and 18, after arthroscopic partial resection, focusing on cellularity, arrangement of collagen fibers, and vascularity of the excised fragments. Furthermore, to report on MRI findings compared to the histological findings in the same region.

## Methods

This study is conformed to the Declaration of Helsinki, as revised in 2000. From 2012 to 2016 six patients, one female and five males, aged 9, 10, 13, 15, 17, and 18, presenting for a symptomatic discoid lateral meniscus at the authors’ Institution, were prospectively scrutinized, enrolled in the study and operated on. At preoperative MRI and at arthroscopic examination, a complete variant was diagnosed in all cases (Watanabe et al., 1979). Furthermore, all patients presented for mechanical symptoms (locking, caching, popping) and/or pain without evidence of meniscal tear at MRI and arthroscopic evaluation. At pre-operative MRI, menisci were classified into three groups according to Hamada et al. (Hamada, 1994):

- Group I: linear, horizontal region of high signal intensity in the substance of the meniscus (4 cases);
- Group II: flattening of the meniscus in addition to regions of high signal intensity in the substance (2 cases);
- Group III: regions of high signal intensity in the anterior segment of the meniscus alone (none).

During each intervention, partial meniscectomy (saucerization) with preservation of a stable peripheral rim was performed to obtain the shape of a normal meniscus. The resected tissue, the central part of the discoid meniscus, was excised with a one-piece technique, anonimyzed, processed in EDTA buffered for 2 h to soften the tissue and fixed in 4% buffered formaldehyde for 24 h. Before proceeding to dehydration each surgical tissue was cut into two fragments and then placed in a series of graded ethanol solutions. Each fragment was then embedded in paraffin with different orientation, either parallel or perpendicular to the articular horizontal plane. From the paraffin blocks, 3-μm thick sections were cut and stained with hematoxylin and eosine, and Masson trichromic. The sections were examined with an Axioskop microscope (Carl Zeiss Light Microscopy, Göttingen, Germany) and photographed with a digital camera (Rt Slider, Diagnostic Instrument Inc., Germany).

## Results

**Fig. 1:**
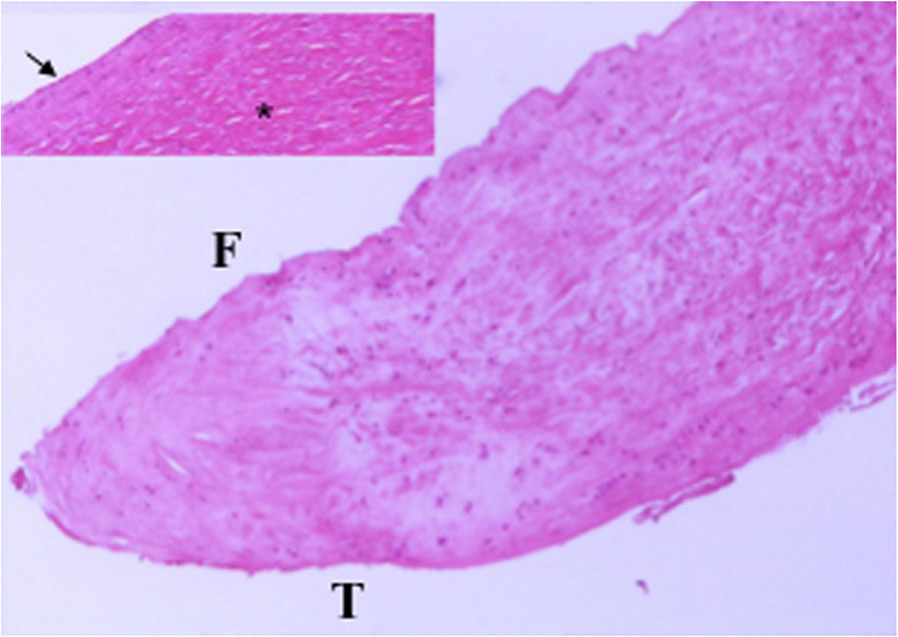
Full-thickness section cut perpendicular to the surface of a discoid lateral meniscus in a 13-year old patient (5× magnification, H&E). Within the fibrocartilaginous tissue, was possible to see a different architecture between the femoral and the tibial surface. In fact, while the tibial surface was smooth and free of irregularities, the femoral surface appeared wavy, with some clefts. Furthermore the orientation of the collagen fibers varied from the periphery to the center of the tissue. While on the periphery the collagen fibers were oriented parallel to the surface, in the inner part of the tissue a random orientation was observed with an interwoven tighter network of collagen fibers. Moreover, in some areas of the tissue, degeneration could be seen, with an extracellular matrix showing a lower intensity of staining with totally disorganized collagen fibers. The upper left box showed the same case at a greater magnification. On the articular side, cells had a more flattened morphology, with the major axis parallel to the surface (arrow). In contrast, cells in the central portion appeared more round. It was possible to see several tears due to tissue damage (asterisk). Cells were scattered inside the different portions of the tissue with some areas of hypo-cellularity or totally devoid of cells as a sign of degeneration

**Fig. 2:**
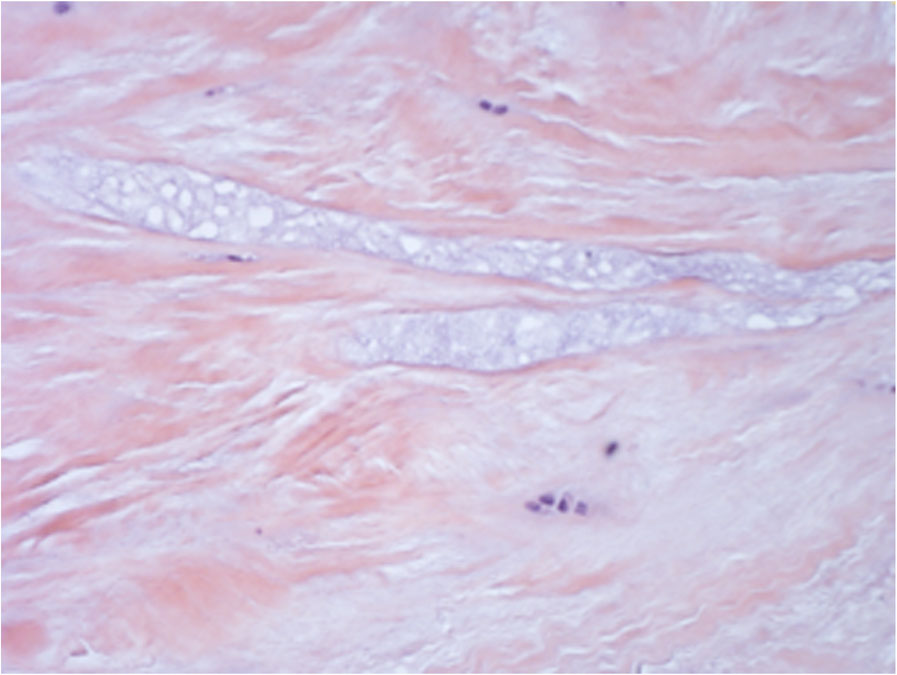
Full-thickness section cut perpendicular to the surface of a discoid lateral meniscus in an 18-year old patient (20×, H&E). The sample showed the same features observed in Fig. 1 but with a more marked aspect of tissue degeneration

Full-thickness sections cut perpendicular to the surface of the meniscus allowed studying the tissue architecture observed from the femoral surface to the tibial surface. A fibrocartilaginous tissue, characterized by fibrochondrocytes embedded in an extracellular matrix composed primarily by water, collagen, and glycoproteins was evident in all cases. However, these elements showed a different distribution and architectural characteristics depending on the different portion of the meniscus. On the periphery of both the tibial and femoral surfaces, the extracellular matrix, generally rich in sulfated glycosaminoglycans, showed a lower density, evidenced by a lower intensity of staining in all cases (Fig. 1). The collagen fibers showed an initial orientation parallel to both the outer surfaces and then, in the inner part, changed their disposition showing an intersection at various angles with an intricately woven arrangement. Furthermore, in all cases, in the central part of the tissue, the collagen fibers were larger and formed a tighter network much more evident than in other portions of the tissue. Within the context of the fibrocartilaginous tissue, it was possible to see several tears due to tissue damage (Fig. 1 asterisk). In addition, in some areas of the tissue it was possible to observe degenerated extracellular matrix with disorganized and less dense collagen fibers (Fig. 1). Examining the surface of the meniscus, both on the femoral and the tibial side, cells had a more flattened, fusiform morphology with the major axis parallel to the surface and were very similar in appearance to fibroblasts (Fig. 1 arrow). In contrast, meniscus cells in the central portion of the tissue appeared with a more round/oval shape, thus very similar to chondrocytes (Fig. 1). The cellular density and distribution were very different from intact semilunar meniscus. The decreasing cell gradient from the surface to the depth of the tissue, described in intact semilunar menisci, was completely absent. On the contrary, the cells were scattered inside the different portions of the tissue with some areas of hypo-cellularity or totally devoid of cells as a sign of degeneration (Figs. 1 and 2). In addition, while the tibial surface appeared mainly smooth, the femoral surface appeared undulated, frayed or in some cases with clefts (Fig. 1). The vascular supply was completely absent in all specimens, but the 18-year old. This sample showed the same features of the other patients but with a more marked aspect of tissue degeneration (Fig. 2).

**Fig. 3:**
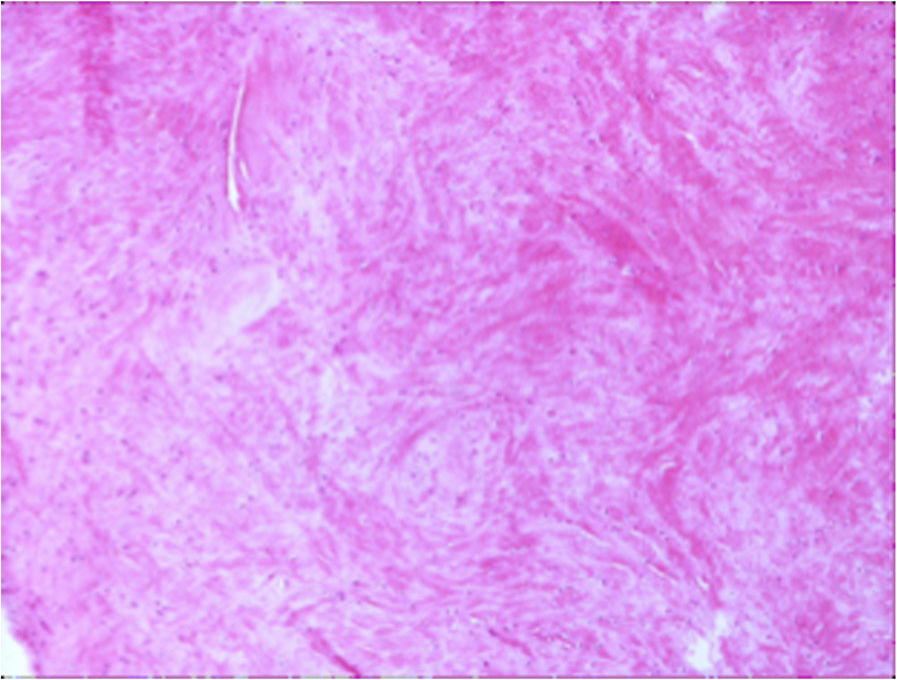
Full-thickness section cut parallel to the surface of a discoid lateral meniscus in a 13-year old patient (10×, H&E). The figure showed a fibrocartilaginous tissue consisting of edematous areas in which round cells associated with thick bundles of collagen fibers distributed with variable orientation

**Fig. 4:**
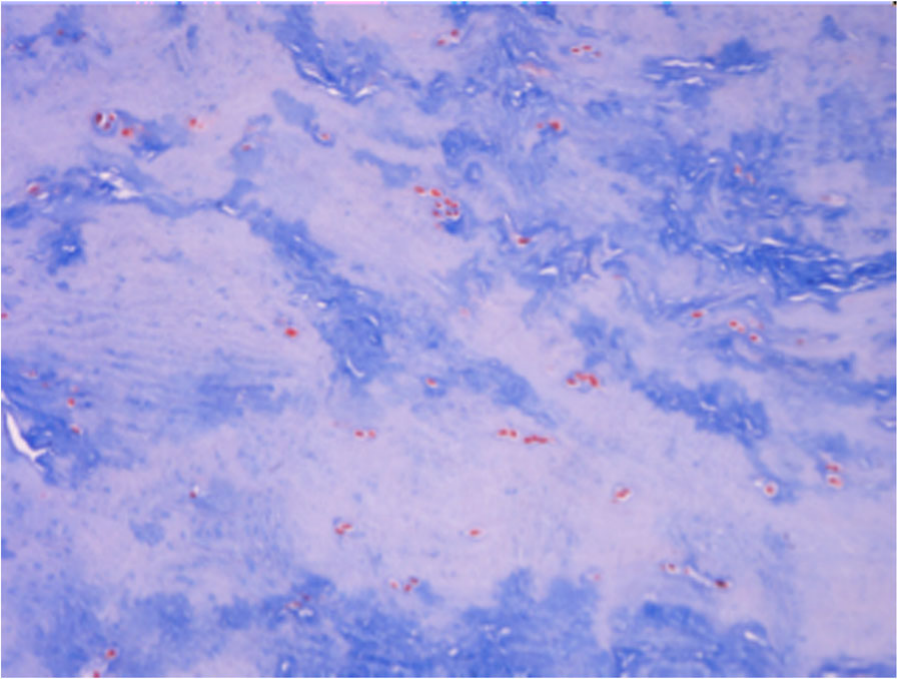
Full-thickness section cut parallel to the surface of a discoid lateral meniscus in a 17-year old patient (20× Masson’s trichrome). Tissue with areas characterized by a marked alteration of the extracellular matrix density clearly highlighted by a non-homogeneous intensity of staining. In these areas, the collagen fibers showed an undefined arrangement, with a loose texture. A drastic reduction or a completely absence of the cellular component was also observed

**Fig. 5:**
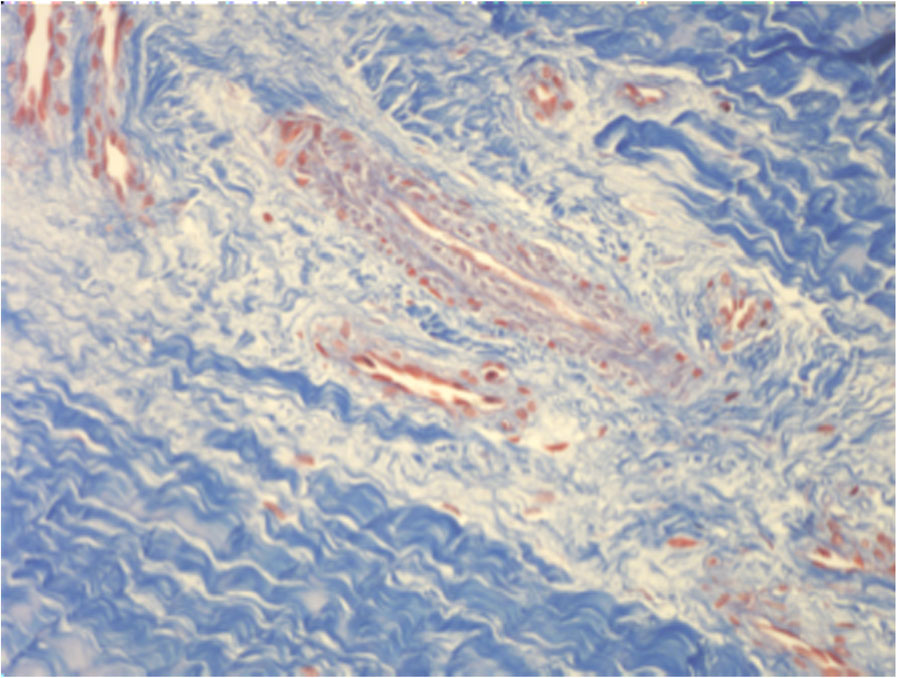
Full-thickness section cut parallel to the surface of a discoid lateral meniscus in an 18-year old patient (10×, Masson’s trichrome). This sample, showed reactive/reparative aspects, represented by areas with numerous blood vessels in which swollen endothelial cells were sometimes found

**Fig. 6:**
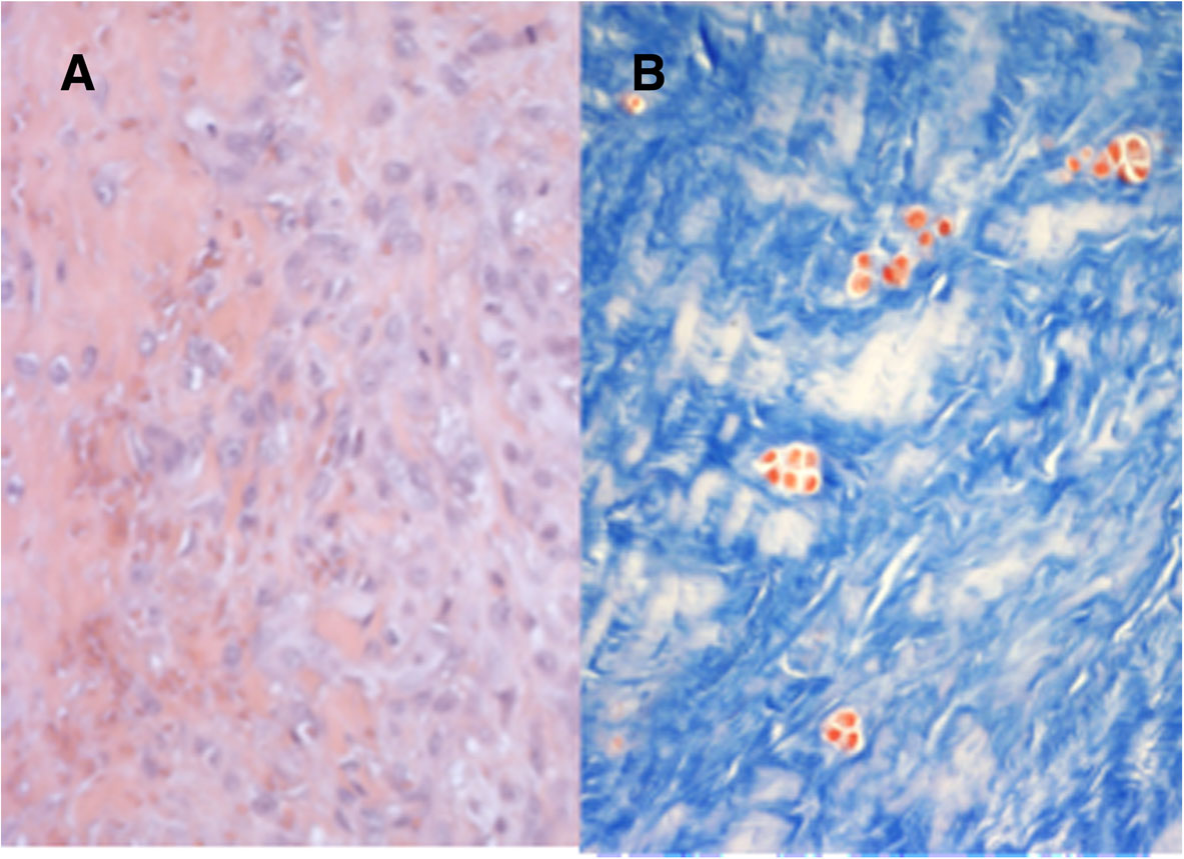
Full-thickness section cut parallel to the surface of a discoid lateral meniscus in an 18-year old patient. **a** (20×, H&E). Fibroblasts gathered in large aggregates associated to the presence of areas of edematous tissue with zones of extravasation of erythrocytes. **b** (20× Masson’s trichrome). Lacunae with cells clusters resembling chondrocytes, characterized by a round shape, were associated to degenerated areas

From a comparison between the sections cut parallel to the articular surface it was possible to see at all ages, on both the femoral and the tibial surface, thick bundles of almost homogeneous size, with variable direction. In this contest, cells with round morphology and a random distribution were present associated with edematous areas (Fig. 3). However, as for the full thickness sections previously described, some areas of the tissues showed a lower density of the extracellular matrix, evidenced by a lower intensity of staining, with disorganized and inhomogeneous aspect. In these areas the collagen fibers were distributed without a definite arrangement, showing a loose texture and the cellular component underwent a drastic reduction or was completely absent. (Fig. 4). Exclusively in the sections obtained from the 18-year old patient, it was possible to see, both in the horizontal and frontal sections, areas with regenerative/reparative aspects characterized by numerous blood vessels covered by swollen endothelial cells, associated with cell aggregates represented by fibroblasts showing an elevated mitotic index. Close to these areas, the presence of edematous tissue was observed in addition to zones of extravasation of erythrocytes (Fig. 5). Moreover, cell clusters inside the lacunae (resembling chondrocytes) with a round shape and larger than the fibrochondrocytes described in normal menisci, were seen associated to degenerated areas (Fig. 5; Fig. 6a and b).

## Discussion

The main finding of the current study is the description of the histologic features of discoid lateral meniscus in a group composed only by children and adolescents (aged 9–18) without a meniscal tear. The main feature observed is severe disorganization of the collagen fibers, irrespectively of the age of the patients. Furthermore, we found no vessels even in the inner part of discoid lateral meniscus in all patients, but the 18-year-old.

In particular, the findings of the current study provide some insights on the vascularity of discoid lateral meniscus. Vascularity of normal meniscus has already been described by Clark et al. (Clark & Ogden, 1983) who reported on a great number of cells and blood vessels during the fetal development of the normal meniscus, that are progressively lost after birth. Even though the meniscus reaches its adult structure between the ages of 10 and 11, also in patients of these ages, blood vessels still could be found throughout the inner parts. In a previous study, Inoue et al. (Inoue et al., 2016) reported on a great concentration of blood vessels in the intercondylar region of complete discoid lateral meniscus in a small group of 6 patients (aged 8 to 17 years). Our results are in disagreement with Clark et al. (Clark & Ogden, 1983) and Inoue et al. (Inoue et al, 2016), in fact we were not able to find any blood vessel in samples from our youngest patients (aged 9 and 10), but only from the 18-year old one.

To the best of our knowledge, there have been only 2 cases of regrowth of a discoid lateral meniscus after surgery, in a 5-year-old girl (Stein et al., 2013) and in an 11-year-old boy (Bisicchia & Tudisco, 2013). In both cases, the authors did not perform histology. However, Bisicchia et al. (Bisicchia & Tudisco, 2013) speculated that the vasculature in discoid menisci should be the same as in normal ones and suggested the blood vessels in the inner part of the meniscus as a possible explanation of the re-growth after surgery. The results of the current study do not support their theory, and question how the re-growth was possible. In fact, we found blood vessels only in the oldest patient, and not in the youngest ones.

To the best of our knowledge, there are only few papers in literature about the histology of discoid meniscus (Atay et al., 2007; Papadopoulos et al., 2009; Cui & Min, 2007; Choi et al., 2017; Inoue et al., 2016). The main limitation of those studies is the inclusion of a great percentage of adults and meniscal tears. Atay et al. (Atay et al., 2007) reported collagen fibers to be fewer and to have a heterogeneous course. Most of their patients were adults, with a mean age of 29 years (range, 5–50 years). All these features were found in all patients, therefore also in the two pediatric ones (5 and 9 years, respectively). This is in agreement with our data showing that the same severe disorganization of the collagen fibers is present in all patients, irrespectively of their age.

Cui et al. (Cui & Min, 2007) reported the femoral surface to be covered by dense and well-arranged thick fibrils, while the tibial surface to show an irregular orientation of fibers. We did not analyze the whole meniscus as Cui et al. (Cui & Min, 2007), but we observed the tibial surface to be flat and smooth while the femoral side to be undulated and often with clefts. We could speculate that the slipping pressure of the femur could easily fray the anomalous tissue observed in the discoid meniscus.

Subsequently, Papadopoulos et al. (Papadopoulos et al., 2009) confirmed the significant disorganization of the circular collagen network in the discoid meniscus with a heterogeneous course of the circumferentially arranged collagen fibers, compared with the normal meniscus with areas of degeneration or osseous metaplasia. The results of their study (Papadopoulos et al., 2009) are biased by the age of their patients. In fact, all of their patients were adults, with a mean age of 30.22 ± 7.61 years (range, 17 to 39 years). Also, in our study, the significant disorganization of the circular collagen network in the discoid meniscus with a heterogeneous course of the circumferentially arranged collagen fibers was observed. We did not find any areas of metaplasia in our younger patients and we speculate that the presence of areas of metaplasia in our oldest patient were due to the progressive meniscal degeneration with ageing and not related to discoid shape itself.

Choi et al. (Choi et al., 2017) in their ultrastructural study found that intact discoid lateral menisci had a more homogenous collagen pattern and greater collagen fibrils number, compared with their torn counterparts. Even in this study, most of their patients were adults (30 subjects included, aged 15 to 58). The results of our study disagree with the findings reported by Chioi et al. (Choi et al., 2017), in fact we observed severe disorganization of the collagen fibers also in intact discoid lateral meniscus.

In agreement with the findings of previous studies (Atay et al., 2007; Papadopoulos et al., 2009; Cui & Min, 2007; Choi et al., 2017; Inoue et al., 2016) we found irregularly oriented collagen fibers in discoid lateral meniscus. On the other hand, in contrast to the results reported by Clark et al. (Clark & Ogden, 1983), there were no blood vessels in the inner part of discoid lateral meniscus in our youngest patients (9 and 10 years old).

Limitations should be acknowledged for this study. A limited number of patients were included due to selection only of children and adolescents. In fact, most of the published studied included also adults (Atay et al., 2007; Papadopoulos et al., 2009; Cui & Min, 2007; Choi et al., 2017). Only Inoue et al. (Inoue et al, 2016) included only children and adolescents and their sample size in comparable to the current study. Our aim was to report on this particular population of patients that may challenge the orthopedic surgeon. We believe that inclusion of patients of all ages to increase the sample size of our study may significantly bias the results and lead to incorrect conclusions.

Histologic analysis was performed only on the resected fragment instead on the whole meniscus. In a previous study, Hamada et al. (Hamada, 1994) resected discoid lateral menisci “en-block” and analyzed the entire meniscus. Given the importance of the meniscus in load distribution, lubrication, and cushioning, the current trend in meniscal preservation surgery, and the long-term outcomes in favor of saucerization over total meniscectomy (Smuin et al., 2017), we believe it is not ethical to resect “en block” an entire meniscus in children and adolescents. Lack of electron microscopy analysis is another limitation of this study, but this microscope is not available at the author’s Institution.

## Conclusions

The findings of our study suggest that discoid lateral meniscus is different from a normal meniscus in terms of vascularity and disorganization of collagen fibers.
